# Efferocytosis of Pathogen-Infected Cells

**DOI:** 10.3389/fimmu.2017.01863

**Published:** 2017-12-22

**Authors:** Niloofar Karaji, Quentin J. Sattentau

**Affiliations:** ^1^The Sir William Dunn School of Pathology, The University of Oxford, Oxford, United Kingdom

**Keywords:** phagocytosis, efferocytosis, cell death, pathogen, bacteria, virus, parasite, inflammation

## Abstract

The prompt and efficient clearance of unwanted and abnormal cells by phagocytes is termed efferocytosis and is crucial for organism development, maintenance of tissue homeostasis, and regulation of the immune system. Dying cells are recognized by phagocytes through pathways initiated *via* “find me” signals, recognition *via* “eat me” signals and down-modulation of regulatory “don’t eat me” signals. Pathogen infection may trigger cell death that drives phagocytic clearance in an immunologically silent, or pro-inflammatory manner, depending on the mode of cell death. In many cases, efferocytosis is a mechanism for eliminating pathogens and pathogen-infected cells; however, some pathogens have subverted this process and use efferocytic mechanisms to avoid innate immune detection and assist phagocyte infection. In parallel, phagocytes can integrate signals received from infected dying cells to elicit the most appropriate effector response against the infecting pathogen. This review focuses on pathogen-induced cell death signals that drive infected cell recognition and uptake by phagocytes, and the outcomes for the infected target cell, the phagocyte, the pathogen and the host.

## Introduction

To maintain and protect themselves, multicellular organisms remove dead and dying cells arising during normal tissue development and function (1) or triggered by infection or sterile inflammation (2). At steady state, even within tissues with high constitutive rates of apoptosis, the number of detectable apoptotic cells is relatively low, indicating a high rate of removal (3–5). Efficient clearance is vital for the constant removal of approximately 10^6^ cells/s undergoing apoptosis in various tissues in adult humans (6). Phagocytosis is defined as engulfment of particulate matter of >0.5–1 µm (7, 8) and is mediated by both professional and non-professional phagocytic cell types. Professional phagocytes are primarily macrophages and immature dendritic cells (DCs) resident in multiple tissues and tissue-infiltrating monocytes, neutrophils, and eosinophils. Non-professional phagocytes such as epithelial cells of mammary epithelium (9) and astrocytes in the brain (10) can also capture and engulf material including dying cells that are present in close proximity within tissue. “Efferocytosis” is a term describing the engulfment by phagocytes of dying and dead cells and their debris (11, 12) and demonstrates features of both conventional phagocytosis and the fluid-phase uptake mechanism macropinocytosis (13–15), resulting in uptake into “spaceous phagosomes” (15). However, although the term efferocytosis distinguishes recognition and engulfment of dead and dying cells from phagocytosis of other objects (12, 16), we are unaware of specific mechanistic differences that discriminate between the two processes. Efferocytosis is mediated by a variety of interactions between the phagocyte and its dying target cell that show substantial redundancy and many soluble and cell surface receptor–ligand interactions defined for phagocytosis are used in efferocytosis (described in more detail below). The initial definition of efferocytosis related to clearance of apoptotic cells (15), but this has more recently been widened to include other modes of cell death (17, 18).

**Figure 1:**
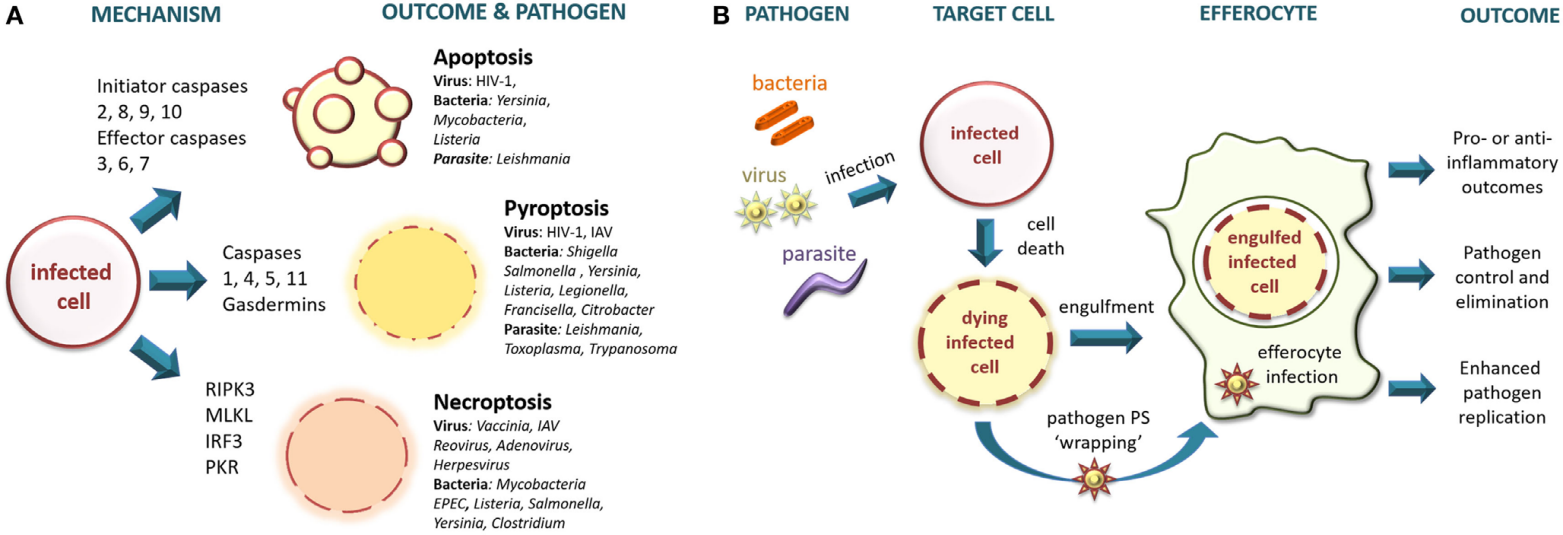
**(A)** Intracellular pathogens trigger regulated modes of cell death. Intracellular bacteria, viruses, and parasites infect target cells triggering different cell death pathways. Apoptosis is a non-inflammatory type of cell death in which the cellular contents remain membrane enclosed, whereas pyroptosis and necroptosis result in compromised membrane integrity leading to the release of intracellular contents that are pro-inflammatory. RIPK3, receptor-interacting protein kinase 3; MLKL, mixed lineage kinase domain-like; IRF3, interferon regulatory factor-3; PKR, protein kinase R; IAV, influenza A virus; EPEC, enteropathogenic *Escherichia coli*. **(B)** Pathogen infection modulates efferocytic outcomes. Intracellular bacteria, viruses, and parasites infect target cells inducing apoptosis. Dying target cells expose eat me signals and may down-modulate don’t eat me signals, leading to uptake and engulfment by efferocytes. Alternatively, the pathogen may escape from the infected cell wrapped in phosphatidylserine (PS)-containing membrane to deploy “apoptotic mimicry” for entry into the efferocyte. Efferocytosis may eliminate the pathogen, or may allow the pathogen to infect the efferocyte in a Trojan-horse type maneuver. The efferocyte will initiate anti-inflammatory or pro-inflammatory signaling depending upon the combined presence of immune-silencing signals (e.g., PS) and pro-inflammatory pathogen-associated molecular patterns and damage-associated molecular patterns.

Cell death has been broadly categorized into accidental (necrosis) and regulated (including apoptosis, pyroptosis, and necroptosis) (17). Accidental cell death occurs during severe physical or chemical insult, such as membrane shearing and rupture *via* extremes of pressure, temperature, osmolarity, pH, or exposure to agents such as detergents and bacterial toxins, and is insensitive to pharmacologic or genetic manipulation. Because accidental cell death results in uncontrolled release of cell contents including damage-associated molecular patterns (DAMPs), it is pro-inflammatory (19, 20). Regulated modes of cell death are implicated in post-embryonic ontogeny, tissue homeostasis, and during infection and immune responses, have a genetically programmed component, and can be modulated by altering pro- and anti-death signals (17). Pathogen infection is variously associated with all forms of regulated cell death (Figure 1A) and necrosis, and the type of cell death induced is directly linked to the type of infecting pathogen, its life cycle and its pathogen-associated molecular patterns (PAMPs) recognized by pattern recognition receptors (PRRs).

## Pathogen-Triggered Cell Death

Apoptosis is a caspase-dependent programmed form of regulated cell death resulting in a series of well-characterized morphologic and molecular changes culminating in membrane blebbing, DNA fragmentation and expression of signals designed to attract phagocytes and trigger engulfment and disposal of the apoptotic cargo (17). Apoptotic cells that are not rapidly efferocytosed become late apoptotic cells with a phenotype related to that of necrosis and are pro-inflammatory (21, 22). Apoptosis may be triggered in various cell types by a variety of pathogens, including intracellular bacteria (23), parasites (24), and viruses (25). Despite having evolved in part as a mechanism to limit pathogen replication and spread, apoptosis may have been subverted to contribute to pathogen survival and disease pathogenesis as discussed below. Pyroptosis is a regulated mode of cell death triggered principally by infection with intracellular pathogens (26, 27) and is linked to inflammasome activation driving caspase-1 or non-canonical caspase-11-triggering of the pore-forming effector gasdermin family (28, 29). Since pyroptosis results in plasma membrane permeabilization and eventual rupture with release of cytoplasmic contents, it has pro-inflammatory outcomes similar to accidental cell death and necroptosis. Necroptosis is triggered by infection with a variety of intracellular pathogens including viruses and bacteria (30, 31). Similar to pyroptosis, necroptosis is also a pro-inflammatory mode of regulated cell death initiated by cell surface receptors but is triggered in a caspase-independent RIPK3-dependent manner and may be modulated by caspase-8 activation toward apoptosis (17). Non-canonical forms of necroptosis activated by IRF3 and PKR-dependent pathways have also been described (31). The different modes of pathogen-initiated cell death and their mechanisms have been recently reviewed [e.g., Ref. (27, 30–33)] and so will not be further discussed here but are summarized in Figure 1A with some examples of pathogens implicated.

## Efferocytic Signals

Phagocytes engage apoptotic cells *via* a defined set of markers termed “apoptotic cell-associated molecular patterns” or ACAMPs (16). ACAMPs include externalized phosphatidylserine, calreticulin, and modified carbohydrates that are recognized by a set of specific receptors and bridging molecules and will be described briefly below. Efferocytosis of dead and dying cells can be divided into four distinct stages (34, 35): (i) Detection of the target cell by release of chemotactic “find me” signals (36, 37) that include lysophosphatidylcholine, CX3CL1 (fractalkine), nucleotides adenosine triphosphate and uridine 5′ triphosphate, and sphingosine 1-phosphate (38–41). (ii) Exposure of “eat me” signals (42, 43), of which phosphatidylserine (PS) exposure on the outer leaflet of the plasma membrane is the best characterized, and although initially described in the context of apoptosis, appears to be shared between all modes of cell death (43–47). However, whereas PS externalization during apoptosis is mediated enzymatically, it becomes accessible during pyroptosis and necroptosis *via* membrane permeabilization. Eat me signaling is counter-balanced by expression levels of “don’t eat me” cell surface molecules such as CD47 (48, 49). Many eat me signals are recognized directly by phagocyte receptors such as members of the T-cell immunoglobulin domain and mucin domain (TIM) family, complement receptors CR3 and CR4, scavenger receptors SRA and CD36, mannose receptor (MR) and integrins α5β3 and α5β5, whereas others require bridging molecules such as collectins, complement C1q, mannose binding lectin, pentraxin3, ficolins, thrombospondin, and milk fat globule protein (MFG-8) for their recognition (18, 42, 50, 51). (iii) Following recognition of eat me signals, receptors such as antibody Fc (52) and complement (53) receptors signal to the cytoskeleton and are directly phagocytic, whereas others such as TIM-4 (54) are implicated only in tethering the target cell. However, a phagocyte will integrate signals from multiple receptors to inform the outcome of whether or not to engulf the target cell (12, 34, 55). (iv) Cellular material is fully internalized *via* cytoskeletal rearrangement of the plasma membrane (5, 12, 35) with processing of the engulfed cell usually (5, 55), but not exclusively (56), leading to its elimination within a phagolysosome-type compartment (57, 58). During the target cell recognition process, phagocytes may further evaluate the target’s chemical composition to estimate the threat posed by its contents and form such as size (59), geometry (60), and topography (61). This assessment determines (i) whether engulfment occurs or is replaced with, for example, neutrophil NETosis, an anti-microbial cell death mechanism whereby neutrophils eject chromatin extracellular traps (62); (ii) the fate of the target cell within the phagocyte; and (iii) whether the clearance process is immunologically silent, such as the efferocytosis of apoptotic cells (11, 63), or stimulates an inflammatory response such as the engulfment of most pathogens, pathogen-infected cells, and necrotic cells (8, 19, 20).

This review will not further consider find me and eat me signals involved in recognition and uptake of dead and dying cells, or the downstream signaling and cytoskeletal changes leading to phagocytic uptake, topics that have been very comprehensively reviewed recently (4, 6, 12, 16, 63–67). Instead, we will highlight recent discoveries regarding phagocyte recognition of cell death triggered by microbial infection and outcomes for the pathogen and infected target cell, the phagocyte, and the host.

## Pathogen Infection Driving Cell Death and Efferocytosis

Infection by intracellular pathogens may lead to cell death by any of the regulated pathways described earlier (see Figure 1A), or by necrotic cell death in the case of some lytic infections. Microbial induction of regulated death is generally considered to be a mechanism evolved to reduce or prevent pathogen replication and spread (33). The beneficial outcomes for the host of pathogen-triggered cell death may comprise: (i) removal of the intracellular environment required for survival and replication; (ii) direct antipathogen effects of released intracellular components; (iii) initiation of an anti-microbial inflammatory response by release of DAMPs and PAMPs; and (iv) uptake and presentation of pathogen antigens by antigen-presenting cells. Induction of cell death may itself be sufficient to reduce and control pathogen replication or may be assisted or mediated by efferocytic mechanisms as has been described for several bacterial and viral pathogens (14). Conversely, some pathogens may use efferocytic mechanisms to invade the phagocyte in a “Trojan-horse” type of manoeuver and thereby perpetuate or enhance replication and dissemination (14, 68). These outcomes are discussed in more detail below and summarized in Figure 1B.

## Efferocytosis in Pathogen Control and Its Subversion by Pathogens

### Bacterial Infection

Gram-negative intracellular pathogenic bacteria including the Enterobacteriaceae *Shigella* (69, 70) and *Salmonella* (71) were initially proposed to induce apoptosis in infected macrophages. However, more recently, this view has been modified to take into account features of pyroptotic cell death including caspase-1 and inflammasome activation (26, 72, 73). Virulent strains of many gram-negative intracellular bacteria have evolved to evade pyroptotic cell death, for example, *Shigella* inhibition of caspase-4 activation (74), testifying to its importance as an innate immune antibacterial mechanism (26, 75, 76). Using an attenuated strain of *Salmonella typhimurium* that constitutively expresses flagellin and, therefore, activates NLRC4, Jorgensen et al. demonstrated that infected pyroptotic macrophages form “pore-induced intracellular traps” that capture bacteria within cellular debris without killing them (77). The bacterium-containing cell debris is then cleared by efferocytic neutrophils that are attracted by find me and eat me signals and kill the bacteria in a ROS-dependent manner (72, 77). Although apoptotic cell death of human monocytes or macrophages infected with wild-type (78–80) or attenuated (81) *Mycobacterium tuberculosis* is associated with reduced bacterial survival, the mechanism was until recently unclear. Apoptosis may impart some cell-intrinsic anti-*M. tuberculosis* activity to macrophages by enclosing the bacilli within apoptotic membrane vesicles, but efferocytosis appears to be an important adjunct mechanism to clear viable bacteria associated with apoptotic macrophages (82, 83). Efferocytosis of apoptotic *M. tuberculosis*-infected macrophages by uninfected macrophages results in their trafficking to a degradative phagolysosomal compartment (83). Similarly, in the zebrafish model, apoptotic *Mycobacterium marinum*-infected granulomatous macrophages were engulfed by neutrophils resulting in their death by oxidative-burst exposure (84). Not only does efferocytosis reduce mycobacterial viability in human cells but also allows cross-presentation by DC of mycobacterial antigens for MHC class-I and CD1 presentation to CD8^+^ T cells in mice, reinforcing adaptive anti-bacterial immunity (85). The importance of macrophage apoptosis as an anti-mycobacterial mechanism contrasts with the finding that some virulent forms of *M. tuberculosis* have evolved to divert apoptotic cell death toward a programmed necrotic pathway, which fails to inhibit bacterial growth and allows bacterial release from the disrupted cell, promoting pathogen dissemination (79, 81, 86, 87). Thus, the type of cell death induced directly influences the outcome for the pathogen.

Bacteria may use efferocytic pathways to escape from elimination or to assist their dissemination using Trojan-horse-type mechanisms in which dying cells carrying viable bacteria are engulfed leading to infection of the efferocyte. After the uptake of *M. marinum* by zebrafish macrophages, the infected macrophage undergoes apoptotic cell death. These apoptotic, infected macrophages are engulfed by other healthy macrophages, driving dissemination of infection *via* efferocytosis to increase granuloma burden and seed secondary granulomas in a manner dependent on the RD1 virulence factor (88). Eat me signals potentially involved in efferocytic mycobacterial infected cell uptake have been partially defined. Blocking of human macrophage cell surface MR using anti-MR antibody or pre-incubation with competitive soluble sugars (mannan and GlcNAc) (89), or blocking TIM-4 (83) significantly reduced the uptake of apoptotic *M. tuberculosis*-infected macrophages by uninfected macrophages. TIM-4 is also implicated in the subversion of efferocytic mechanisms by the gram-positive bacterial pathogen *Listeria monocytogenes*. The bacterium is phagocytosed by macrophages but avoids elimination by escaping the phagosome using the pore-forming toxin listeriolysin O (LLO) and recruits actin to drive cell-to-cell spread (90). The bacterium wraps itself in vesicles derived from the LLO-damaged host cell plasma membrane that exposes PS, which are in turn recognized by TIM-4 on healthy macrophages, leading to bacterial uptake and infection of a new host cell (91). Infection of a mouse strain lacking TIM-4 expression resulted in impaired bacterial growth, thus emphasizing its role *in vivo*. An efferocytic Trojan-horse mechanism of dissemination using neutrophils as a cellular vector is proposed for *Chlamydia pneumoniae* (92) and *Yersinia pestis* (93). In mice, both bacteria are initially phagocytosed by neutrophils at the site of inoculation, but the bacteria survive and, in the case of *Y. pestis*, replicate within the neutrophils. Subsequent infected neutrophil apoptosis and PS exposure triggered efferocytosis by macrophages, which were permissive for replication of both bacteria but elicited an anti-inflammatory cytokine response, potentially limiting anti-bacterial activity (92, 93). Macrophage recognition of *C. pneumoniae* in the context of apoptotic cells was shown to be important for bacterial replication, since inhibition of efferocytosis using annexin-V reduced macrophage infection (92).

### Viral Infection

There is limited work on the role of efferocytosis in controlling viral replication. Influenza A virus infection of HeLa cells resulted in their apoptosis and rapid efferocytic engulfment and transit into phagosome-like structures within murine alveolar macrophages (94). The outcome of this was to limit viral release in the culture, suggesting that this may be a mechanism for suppressing replication *in vivo* (95). Of interest, the authors demonstrated that the eat me signals implicated in this efferocytic uptake were a combination of plasma membrane PS exposure and desialylation of surface glycans on the infected cells (96), consistent with other studies suggesting that loss of cell surface sialic acid during apoptosis is a novel eat me signal (97).

Recently, Baxter et al. observed that recognition and uptake of HIV-1-infected CD4^+^ T cells by human monocyte-derived macrophages led to enhanced macrophage infection when compared to incubation of these cells with cell-free virus (98). Macrophage infection by this cell-to-cell route was high multiplicity allowing robust infection even by weakly-macrophage–tropic viral strains (98). Although infected apparently healthy cells were weakly selectively captured, the strongest eat me signal came from dying HIV-1-infected cells, implying efferocytic signals. However inhibitors of PS–receptor interactions and other apoptotic cell death recognition receptor–ligand interactions failed to significantly reduce infected T-cell uptake, suggesting alternative signals that have yet to be defined (98). Macrophage phagocytosis of simian immunodeficiency virus-infected CD4^+^ T cells occurs in the macaque model, suggesting that this mode of viral spread may have *in vivo* relevance for immunodeficiency viruses, although it is unclear if the macrophages were productively infected or simply harbored infected efferocytosed cells (99, 100). This raises the general caveat that phagocytes may take up pathogen-infected cells giving the appearance of infection but without necessarily undergoing productive infection themselves (68). Thus, astrocytes, long proposed to undergo an atypical HIV-1 infection in the brain but which lack the primary HIV-1 receptor CD4, are phagocytic and engulf dying HIV-1-infected cells leading to markers of viral infection but are resistant to viral entry and infection (101). Finally, in an interesting twist to this paradigm, human papilloma virus (HPV) appears to have subverted efferocytosis to facilitate viral persistence *in vivo*. Efferocytosis of HPV-infected cervical cancer cells by primary human fibroblasts (102) led to expression of the HPV E6 gene within the fibroblasts and elicitation of a tumorigenic phenotype with implications for viral persistence (103).

While not formally efferocytosis, apoptotic cell mimicry achieved by the incorporation of PS into viral envelopes is a related phenomenon that has been described as enhancing infectivity for several enveloped virus families (104, 105) including HIV-1 (106), vaccinia virus (107), and lentiviral vectors pseudotyped with multiple viral envelope glycoproteins (105, 108). Non-enveloped picornaviruses also use this strategy, effected by wrapping themselves in PS-containing vesicles during cellular exit (109, 110). PS incorporated into the viral envelope during budding is recognized by a multitude of PS receptors on the target cell including TIM-1 and TIM-4 and TAM tyrosine kinase receptors TYRO3, AXL, and MER (11, 105, 108, 111) and may be further enhanced by bridging molecules such as MFG-E8 (105). Apoptotic mimicry has the major advantage of compromising pro-inflammatory programs by activating anti-inflammatory signaling cascades, which would otherwise trigger innate and adaptive immune responses against the invading virus, reducing viral replication *in vivo* (112). This immune evasion strategy is also used to good effect by parasites (see below).

### Parasite Infection

*Leishmania* infection is transmitted to man by the sandfly, recruits a rapid neutrophil influx to the site of parasite entry, and replicates primarily within macrophages. The *Leishmania major* inoculum consists of a mixture of apoptotic and viable parasites, and depletion of apoptotic parasites reduces *in vivo* infectivity in a mouse model, proposed to be a consequence of loss of the anti-inflammatory TGF-β signal imparted on macrophages by the PS-expressing parasites (113). Using intravital microscopy of infected sandfly bites in mouse ear, it was observed that neutrophils are rapidly recruited to the site of the bite and engulf the parasites, many of which remain viable and infectious (114). *Leishmania* uptake by neutrophils can delay or accelerate neutrophil death in a manner dependent upon the experimental system. *In vitro* studies demonstrate that apoptosis of neutrophils is delayed for up to 2 days by *L. major* infection, thereby potentially serving as intracellular survival vectors for the parasites, during which time the neutrophils release MIP-1β, which attracts monocytes and macrophages (115). However, by contrast with *in vitro* studies, *Leishmania*-infected neutrophils analyzed *ex vivo* showed enhanced expression of PS, indicating accelerated apoptosis and potentially serving as an eat me signal for macrophage and DC uptake (116). Regardless of the underlying mechanism, macrophages may then efferocytose-infected apoptotic neutrophils becoming infected themselves in the process, the Trojan-horse mechanism (113). An alternative scenario, imaged by intravital microscopic analysis, is that rather than carrying parasites into the macrophage by efferocytosis, neutrophils may release viable parasites into regions densely populated by macrophages for subsequent macrophage engulfment and infection (114). Interestingly, in this study, depletion of neutrophils reduced *L. major* infection in mice (114), consistent with the idea that efferocytosis of apoptotic neutrophils imprinted an anti-inflammatory TGF-β signal on the neutrophils, preventing effective parasite elimination (115). A similar finding was also obtained when apoptotic neutrophils were engulfed by *L. major*-infected macrophages that produced the anti-inflammatory mediators TGF-β and prostaglandin PGE2 (117). However, these results must be evaluated in the context of the strain of mouse, the species of parasite, and the timing of macrophage encounter with apoptotic neutrophils in comparison with their encounter with the parasite. By contrast with the BALB/c mice used in the study above, engulfment of neutrophils by *L. major*-infected macrophages from parasite-resistant C57BL/6 mice reduced parasite load, concomitant with the secretion of TNF that most likely antagonized the anti-inflammatory signals released by uptake of apoptotic neutrophils (117, 118). Moreover, parasite killing may be parasite species dependent, since neutrophils from parasite-susceptible BALB/c mice triggered macrophage killing of *Leishmania amazonensis* and *Leishmania braziliensis* (119, 120). Finally, efferocytosis of apoptotic neutrophils by macrophages from resistant C57BL/6 mice 3 days prior to encounter with *L. major* led to enhanced permissivity to the parasite (121), a result that contrasts with studies in which infection took place prior to neutrophil exposure. Thus, in summary, whether efferocytosis of dying neutrophils results in advantageous or deleterious consequences for the parasite is complex and context dependent.

*Trypanosma cruzi* infection induces lymphocyte apoptosis in both experimentally infected mice (122, 123) and infected humans (124), and disease severity correlated with the degree of *ex vivo* apoptosis observed (124, 125). Mouse experiments support the concept that phagocyte uptake of apoptotic T lymphocytes results in the establishment of an anti-inflammatory response dictated by TGF-β and prostaglandin PGE2 that fuels parasite persistence and disease (126). Treatment of *T. cruzi* infected mice with inhibitors of apoptosis reversed the anti-inflammatory phenotype and reduced *ex vivo* parasite replication (123, 127), consistent with efferocytosis of apoptotic cells reducing macrophage anti-parasite activity and enhancing parasite persistence and disease.

## Immune Consequences of Infected Cell Efferocytosis

The outcome for the phagocyte of engulfment of an infected dying cell is influenced both by the infecting pathogen and by the mode of death elicited in the target cell. PS exposed on apoptotic cells delivers an anti-inflammatory signal that is associated with defined receptor and signaling pathways and the production of regulatory cytokines such as TGF-β and IL-10 (11, 66) and is essential for rapid removal of apoptotic cells to avoid inflammatory and potential autoimmune consequences (19, 63). However, this is in the context of uninfected cells undergoing homeostatic apoptosis and immune-silent clearance. As described earlier, pathogen infection may induce more pro-inflammatory types of cell death *via* release of DAMPs and components of the pathogen present within the infected dying cell act as PAMPs to signal a pro-inflammatory response through PRRs (128). Thus, the phagocyte must integrate the pro- and anti-inflammatory signals to initiate the appropriate outcome, resulting in pathogen containment or clearance. Due to this complexity, experiments to probe the effects of specific pathogen infections of target cells on phagocyte pro- and anti-inflammatory programs are challenging to design and interpret. However, some information is available, particularly with regard to outcomes of efferocytosis of bacterial infection in the context of apoptotic cells. Torchinsky et al. (129) demonstrated that the combination of apoptotic and TLR-4-based signals presented to DCs by apoptotic neutrophils or B cells associated or not with *E. coli* or LPS triggered the release of TGF-β and IL-23 in the context of IL-6, a cytokine pattern, which favors the induction of Th17 effector Th cells. Th17 cells secrete the cytokine IL-17, which is important for the recruitment of neutrophils to resolve bacterial infections, and the combination of apoptotic cells and bacterial PAMPs was optimal for Th17 induction in the context of the model *Citrobacter rodentium* infection of mouse gut (129). LPS alone failed to induce biologically active TGF-β and also induced high levels of IL-12, favoring a Th1-type response rather than Th17-type response, an outcome that would be suboptimal for extracellular bacterial clearance. Similarly, comparison of DC-mediated efferocytosis of an uninfected or *E. coli*-infected macrophage line induced to undergo apoptosis resulted in distinct outcomes (130). DC efferocytosis of infected apoptotic cells showed increased CD86 and CCR7 expression associated with an enhanced migratory capacity compared to uninfected apoptotic cells and enhanced production of IL-6, TGF-β, and IL-23, indicative of Th17 differentiation capacity (130). This again suggests that combination of pathogen and dying cells integrates signals to elicit the most appropriate immune outcome to control the specific pathogen. However, infection associated with apoptosis may also lead to misdirected adaptive immunity resulting in autoimmune outcomes. Thus, using apoptotic B cells infected with *L. monocytogenes*, Campisi et al. showed that the combination of stimuli present within the phagocyte resulted in presentation of self-antigens in the context of a pro-inflammatory environment (131). Using an *in vivo* murine model, this translated into Th17-induced colitis in the context of a *C. rodentium* bacterial infection, confirming the potentially deleterious effects of co-presentation of apoptotic and inflammatory infection signals by DC.

The most obvious implication of efferocytic uptake of virally infected dying cells is in cross-presentation, since CD8^+^ T-cell priming against viral infections requires access of viral antigens to the MHC class-I processing and presentation apparatus. While viruses that infect antigen-presenting cells do this directly by cytoplasmic expression of their antigens, induction of immune responses to viruses that do not productively infect DCs rely upon efferocytosis of infected apoptotic cells followed by cross-presentation. The first demonstration of this was in the context of influenza virus infection of monocytes that led to their apoptosis and uptake by DCs, driving efficient CD8 T-cell priming against influenza antigens (132). Since then, multiple studies have reported on cross-presentation of viral antigens by efferocytic uptake of dying cells infected with vaccinia virus, HTLV-1, measles virus, CMV, and EBV (133, 134). Similar observations have been made for a series of intracellular bacterial pathogens including *L. monocytogenes* and *M. tuberculosis* (133). Although much of the cell biology of cross-presentation has been defined, what remains to be addressed is how the combination of cell death and pathogen-triggered signals influence the outcome of cross-presentation, as for example has been dissected for T-helper cell responses (128). The restriction factor SAMHD1 renders DC relatively resistant to HIV-1 infection and limits DC activation and antigen presentation (135). However, as recently demonstrated by Silvin et al. (136), DCs are heterogeneous with respect to viral infection and, while CD1c^+^ DCs are sensitive to HIV-1 and influenza virus infection resulting in DC death, CD141^+^ DCs are resistant. The authors provide evidence that in the absence of direct infection, CD141^+^ DC acquires viral antigen by efferocytosis of dying virus-infected cells including CD1c^+^ DC, allowing efficient cross-presentation. Also relevant to cross-presentation of infected dying cells, cells dying by necroptosis, rather than by necrosis or apoptosis, trigger a RIPK1-dependent NFκB transcriptional program-directing release of inflammatory cytokines that enhance cross-priming of CD8^+^ T cells by DC (137). Although the cells in this instance were not infected, the relationship between this mechanism and infections driving necroptosis is obvious and raises questions regarding the ability of pathogens to modulate or suppress NFκB activation and other pro-inflammatory programs in dying cells. HIV-1 infection is a weak trigger of type-I interferon responses in macrophages, considered in part to result from “shielding” of viral nucleic acid PAMPs from intracellular sensors (138, 139), although early entry events can elicit low interferon levels (140). HIV-1 infection of CD4^+^ T cells leads to their death by apoptosis (141) during productive infection or pyroptosis during abortive infection (142), and *in vivo*, there is likely to be a combination of these types of death associated with infection. Using model *in vitro* systems, Lepelley et al. showed that HIV-1-infected CD4^+^ T cells elicit robust type-I interferon release from plasmacytoid DCs that is partially elicited by TLR-7 sensing of viral RNA (143) but potentially also by DAMPs released from infected dying T cells. Thus, cell-associated viral PAMPs appear to elicit a stronger innate immune anti-viral response than the virus alone.

## Concluding Remarks

It is evident that efferocytosis is both an essential element of tissue homeostasis and a mechanism for elimination of intracellular pathogens. However, as described earlier, subversion of efferocytic mechanisms *via* (i) the Trojan-horse type of strategy, (ii) cell-free microorganisms expressing or hijacking PS-containing membrane, and (iii) triggering of anti-inflammatory programs in macrophages by efferocytosis of apoptotic cells contributes to immune evasion and pathogen persistence. Therapeutic intervention in efferocytic pathways may well be a rational approach to reducing infection by certain pathogens, but care must of course be exercised to avoid perturbing the fine balance between homeostasis and deleterious inflammation. *In vitro* studies demonstrated a reduction in HIV-1 infectivity of macrophages in the presence of soluble recombinant annexin-V, suggesting a mechanism to target this viral reservoir (144). The anti-PS monoclonal antibody Bavituximab has been used in a number of clinical trials as an anti-cancer agent, and its use in targeting viral infections such as HIV-1 (145), Pichinde virus (as a model for Lassa fever virus), and CMV (146) has been investigated. Aside from directly targeting pathogen replication, chronic infections such as HIV-1 (147) and HCV (148) are associated with long-term inflammatory outcomes that predispose to disease even in the context of suppressive anti-viral regimens. Chronic inflammation in HIV-1 infection is linked to acute inflammatory events in the gut-associated lymphoid tissue (GALT) initiated by massive HIV-1 infection and death of CD4^+^ T cells (149) that predispose this tissue to translocation of microbial products from the lumen (150). Excessive CD4^+^ T-cell apoptotic death may saturate GALT efferocytic capacity driving neglect of apoptotic cells by phagocytes leading to secondary necrosis, a type of cell death associated with tissue infiltration of monocytes and neutrophils that may mediate further tissue damage and is linked to chronic inflammatory autoimmune conditions (22). Moreover, abortive HIV-1 infection of tissue CD4^+^ T cells has been implicated in pyroptotic cell death (142), which might directly promote pro-inflammatory programs in phagocytes. Whether modulating efferocytosis in conditions such as this may influence the inflammatory outcome is a question that requires attention. Very recently, it has been demonstrated that efferocytosis during drosophila development can reprogram macrophages *via* JNK signaling to increased expression of the damage receptor Draper for robust responses to subsequent tissue injury or infection (151). This type of priming leading to innate immune “memory” deserves further investigation and may potentially be targeted for either anti-pathogen or anti-inflammatory outcomes in the clinic.

## Author Contributions

NK and QS conceptualized the article. NK wrote and revised the first draft. NK and QS revised and edited subsequent drafts.

## Conflict of Interest Statement

The authors declare that the research was conducted in the absence of any commercial or financial relationships that could be construed as a potential conflict of interest.
